# Do enlarged white matter perivascular spaces reflect brain clearance dysfunction? Insights from intrathecal contrast-enhanced MRI

**DOI:** 10.1007/s00234-025-03877-7

**Published:** 2025-12-26

**Authors:** Vanja Cengija, Per Kristian Eide, Geir Ringstad

**Affiliations:** 1 Oslo University Hospital, Oslo, Norway; 2 University of Oslo, Oslo, Norway

**Keywords:** Perivascular spaces, Glymphatic function, Cerebrospinal fluid, GMRI, MRI contrast agent

## Abstract

**Purpose:**

Visible perivascular spaces of cerebral white matter at magnetic resonance imaging (MRI) were in several studies proposed to be an integral part of brain-wide perivascular clearance pathways, and their enlargement could therefore serve as markers of perivascular clearance dysfunction. We studied whether MRI-visible perivascular spaces in subcortical white matter communicate with subarachnoid cerebrospinal fluid (CSF).

**Methods:**

MRI-visible perivascular spaces of the basal ganglia served as controls. Intrathecal 0.5 mmol gadobutrol was utilized as CSF tracer, and T1-weighted MRI was performed before, and at multiple time points after (3, 6, 24 and 48 h) injection. Perivascular spaces with diameter ≥ 2 mm were included in the analysis, and a circular region of interest was placed manually within one perivascular space and in adjacent brain parenchyma of each region.

**Results:**

The study included 27 symptomatic individuals undergoing clinical work-up of various CSF circulation disorders, but in whom no treatable condition was found. Perivascular spaces of both white matter and basal ganglia enhanced with intrathecal gadobutrol, confirming CSF exchange with perivascular spaces. While perivascular spaces of basal ganglia enhanced most with peak at 6 h (233.2% [34.9 to 431.5%]) (*p* < 0.01), coinciding with peak enhancement in subarachnoid CSF, perivascular spaces of white matter enhanced less and more slowly with peak at 48 h (159.1% [37.9 to 280.3%]) (*p* < 0.01).

**Conclusions:**

The various degrees of CSF exchange with MRI-visible subcortical perivascular spaces suggest these may be dilated for different reasons, therefore questioning their validity as markers of perivascular clearance function.

**Supplementary Information:**

The online version contains supplementary material available at 10.1007/s00234-025-03877-7.

## Introduction

Subpial perivascular spaces were shown in rodent studies to represent conduits for clearance of potentially neurotoxic solutes from the brain interstitial fluid [1]. This was enabled by CSF influx from the brain surface in Virchow-Robin perivascular spaces along arteries penetrating into cerebral cortex followed by exchange with interstitial fluid and subsequent perivascular drainage. Cortical perivascular spaces are practically never visible at magnetic resonance imaging (MRI) [2], but it was hypothesized that MRI-visible perivascular spaces in cerebral white matter may represent a noninvasive imaging marker of downstream clearance obstruction in the cerebral cortex [2–5]. However, perivascular spaces in cerebral white matter are shown to most typically surround deep medullary arteries [6, 7], which are much less abundant than cortical arteries and are specialized for supply of the outer ~ 2/3 of cerebral white matter. Perivascular spaces of cortical arteries are not directly connected with those surrounding medullary arteries [8]. Therefore, even though load of MRI-visible perivascular spaces in cerebral white matter is associated with conditions where impaired perivascular clearance may be instrumental, including age, cerebral amyloid angiopathy, dementia and hypertension [3, 9–12], other factors than cortical clearance dysfunction may cause enlargement of perivascular spaces in cerebral white matter.

In this study, we investigated the long-term dynamics of CSF enrichment in MRI-visible perivascular spaces in cerebral white matter and adjacent parenchyma. The primary hypothesis was that MRI-visible perivascular spaces are in direct continuity with the brain surface and would therefore enrich homogeneously with CSF tracer, preceding enrichment in adjacent parenchyma. For this, we utilized an intrathecal MRI contrast agent as CSF tracer and obtained consecutive, standardized MRI in a cohort of close to healthy subjects. CSF tracer enrichment in MRI-visible perivascular spaces and parenchyma of the basal ganglia were assessed for comparison.

## Materials and methods

### Study design

The study design was prospective, enrolling consecutive patients who were undergoing intrathecal contrast enhanced MRI as a part of an extensive clinical work-up for suspected CSF disorders. The current study cohort includes patients in whom no CSF disturbance was identified, and no treatment advocated. These subjects can therefore be considered close to healthy, even though they were symptomatic. All study subjects have been included as reference patients in other publications [13–15].

### MRI protocol

MRI scans were performed in a 3 Tesla Philips Ingenia MRI scanner. Standardized T1 MRI acquisitions were performed at multiple time points pre and post intrathecal injection of 0.5 mmol gadobutrol (0.5 ml of 1.0 mmol/ml gadobutrol; Gadovist, Bayer Pharma AG, Berlin, Germany). The injection procedure was performed by an interventional neuroradiologist at morning time and has been described previously [16, 17]. Post-contrast T1 scanning was performed at the time points 3 h, 6 h (Day 1), 24 h (Day 2) and 48 h (Day 3) after contrast injection, respectively. All study subjects remained in supine position until last scan at Day 1, and patients were allowed to move without restrictions after the last scan at Day 1.

Sagittal 3D T1-weighted scans were acquired using the following imaging parameters: Repetition time = ‘shortest’ (typically 5.1 ms), echo time = ‘shortest’ (typically 2.3 ms), flip angle = 8 degrees, field of view = 256 × 256 mm and matrix = 256 × 256 pixels (voxel size 1 mm) [17]. 

3D T2-weighted scans were performed with following imaging parameters: repetition time = 2500 ms, echo time = 331 ms, field of view = 250 × 250 mm and matrix = 250 × 250 pixels (voxel size 1 mm).

Pre-contrast, we also obtained a sagittal 3D FLAIR volume acquisition with the following imaging parameters: repetition time = 4,800 ms; echo time, “shortest” (typically 318 ms); inversion recovery time, 1,650 ms; field of view, 250 × 250 mm; and matrix, 250 × 250 pixels (voxel size 1 mm).

Slice orientation of image stacks was defined using an automated anatomy recognition protocol based on landmark detection in MRI data (SmartExam™, Philips Medical Systems, Best, The Netherlands) for each time point to secure consistency and reproducibility of the MRI slice placement and orientation.

### Image analysis and post processing

MRI-visible perivascular spaces with a diameter of at least 2 mm in diameter were included in the analysis, while those with diameter less than 2 mm were left out to limit the effect of partial volume averaging given a voxel size of 1 mm. We assessed burden of MRI-visible perivascular spaces using T2-weighted images according to a previously reported visual rating scale [18, 19]. Here, perivascular space count is assessed from the axial slice with most perivascular spaces in the cerebral white matter and basal ganglia. Perivascular space burden in the region of basal ganglia and cerebral white matter was scored separately as Score 0 (no MRI-visible perivascular space), Score I (1–10), Score II (10–20), Score III (20–40) and Score IV (over 40). FLAIR was used to differentiate MRI-visible perivascular spaces from lacunar infarcts by the finding of gliosis surrounding the latter.

T1 signal intensity was in each subject measured at multiple time points within one MRI-visible perivascular spaces with diameter ≥ 2 mm located in basal ganglia and in cerebral white matter, respectively. A circular region of interest (ROI) was manually adapted to fit within the perivascular space and carefully placed to avoid adjacent brain tissue. tissue. Typical size of the ROI was up to 1 mm^2^. Examples from basal ganglia are shown in Supplementary Figs. 1 and 2, and from subcortical white matter in Supplementary Figs. 3 and 4. An additional ROI of similar size was placed in the immediate adjacent brain parenchyma, as well as outside the nearest brain surface to assess for local CSF tracer enrichment. The shortest distance to the brain surface was measured for all perivascular spaces included in the ROI analysis.

To adjust for baseline changes at the MRI greyscale that can occur between acquisitions, we normalized the images by dividing the T1 signal unit for each time point by the T1 signal unit of a ROI in the posterior part of the orbit (retrobulbar fat). Changes in normalized T1 signals during the timeline of the study are given as percentage change from baseline and express enhancement of the intrathecal contrast agent, i.e. CSF tracer enrichment. At low tracer (contrast agent) concentrations, the relation between the concentration and SI changes can be considered to be linear [20].

To assess the relative tracer enrichment in an MRI-visible perivascular space versus nearby brain parenchyma we determined the ratio of signal intensity change in perivascular space and nearby brain parenchyma after 24 h and 48 h, respectively. As the water fraction of the extracellular space in brain tissue has been estimated to constitute 20%, a ratio of five in T1 signal increase in perivascular space (~ 100% water) versus extracellular space (~ 20%) [21] is considered to indicate roughly equal tracer concentrations in those two compartments. A ratio above five thus implies a higher tracer enrichment in the perivascular space than in adjacent tissue, and vice versa.

### Statistics

Continuous data were presented as mean ± standard deviation (SD). The repeated measurements of percentage change in normalized T1 signal over time were analyzed using linear mixed models. These models employed maximum likelihood estimation with a subject-specific random intercept and distinct residuals, varied by observation time or group as appropriate. Using the estimated marginal means derived from the statistical model, differences between groups at each time point were tested. Results were presented as mean with a 95% confidence interval (CI). The correlation between continuous variables was assessed using Pearson’s correlation coefficient. Statistical significance was accepted at the 0.05 level, using only 2-tailed P values.

### Data availability

The data presented in this work is available on reasonable request.

## Results

### Patient cohort

The study included 27 participants; individual demographic information and characteristics of perivascular spaces are presented in Table 1. MRI-visible perivascular spaces with diameter ≥ 2 mm were found in the basal ganglia in 24 participants and in cerebral white matter in 15 participants.

**Table 1:** Demographic information of patient cohort and characteristics of the perivascular spaces in basal ganglia and subcortical white matter

	Demographic information			Characteristics of perivascular spaces					
				Basal ganglia			Subcortical white matter		
Patient	Age (yrs)	Sex	BMI (kg/m^2^)	PVS-score	Shape	Dimension (mm)	PVS-score	Shape	Dimension (mm)
1	39	F	41.0	1	linear	2	2	linear	1
2	34	F	24.2	1	oval	2	3	oval	1
3	39	F	27.8	1	oval	1	2	linear	1
4	47	M	25.9	1	linear	2	2	linear	2
5	32	M	24.9	1	oval	2	2	linear	2
6	47	F	24.6	1	linear	3	2	linear	2
7	44	F	34.1	2	linear	5	4	linear	2
8	37	F	32.6	1	linear	3	3	linear	1
9	64	M	25.2	1	oval	2	3	linear	2
10	36	F	26.8	1	linear	1	2	linear	2
11	23	M	24.3	1	oval	2	2	linear	1
12	33	F	19.6	1	linear	2	2	linear	2
13	48	F	24.2	1	linear	1	2	linear	1
14	44	M	27.5	1	linear	2	2	linear	3
15	25	F	31.1	1	linear	2	2	linear	1
16	30	F	23.4	1	linear	2	2	linear	1
17	23	F	24.1	1	linear	2	4	linear	2
18	28	F	24.1	2	oval	2	3	linear	1
19	31	F	37.0	1	linear	2	2	linear	2
20	28	F	28.4	1	linear	2	3	linear	1
21	37	F	35.4	1	linear	3	4	linear	3
22	50	M	26.0	1	oval	4	1	linear	1
23	32	F	28.7	1	linear	2	3	linear	1
24	35	F	22.4	2	oval	2	3	linear	2
25	41	F		1	oval	3	2	linear	2
26	71	F	23.0	2	oval	3	4	linear	2
27	55	F	32.8	1	oval	4	2	linear	2

*F* Female, *M *Male, *BMI *Body mass index, *PVS *perivascular space

### CSF tracer enrichment in PVS of basal ganglia and cerebral white matter

**Fig. 1:**
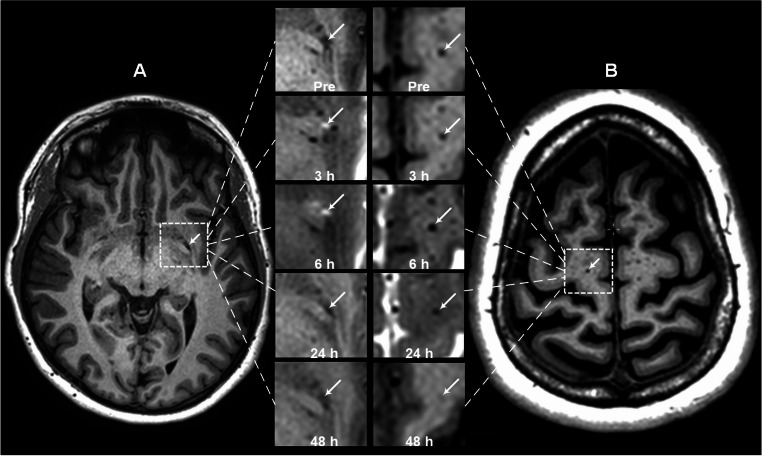
MRI-visible perivascular spaces (PVS).T1-weighted MRI zoomed in on a section (stippled square) of basal ganglia (upper row) and subcortical white matter (lower row). An MRI-visible PVS in each region is pointed out with arrows. A circular region of interest was manually adapted within each PVS at each time point, allowing for assessment of percentage T1 signal increase, where increased T1 signal indicates increased tracer enrichment.

**Fig. 2:**
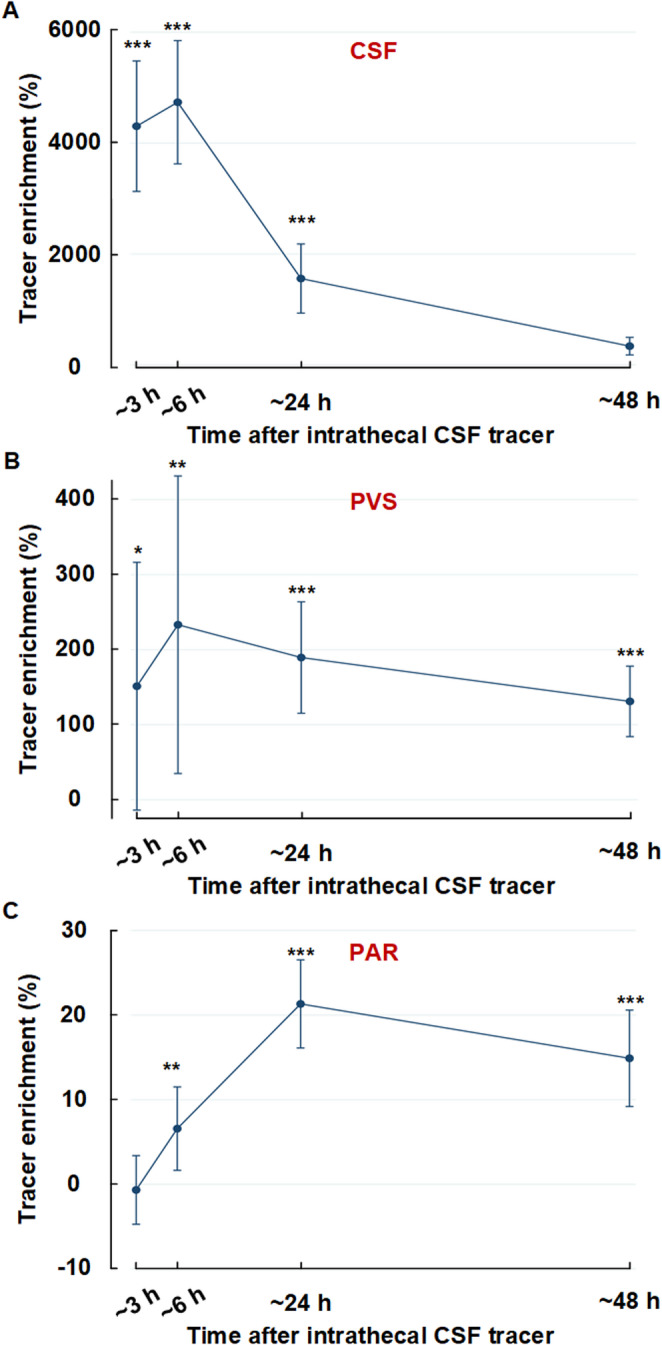
Enrichment of tracer in basal ganglia from subarachnoid CSF space. Tracer enrichment in basal ganglia with regions of interest in (**A**) CSF nearby brain surface towards basal ganglia, (**B**) perivascular spaces (PVS) within basal ganglia and (**C**) brain parenchyma (PAR) nearby PVS. The significant differences in tracer enrichment as compared to pre-contrast are indicated: ^*^P<0.05, ^**^P<0.01,^***^P<0.001 (mixed model analysis). Peak enhancement in subarachnoid CSF and PVS of basal ganglia occurs early and coincides in time, which may indicate a free communication between those two compartments. Tracer enrichment in adjacent brain parenchyma (PAR) is much more limited and peaks at 24 hours (h)

**Fig. 3:**
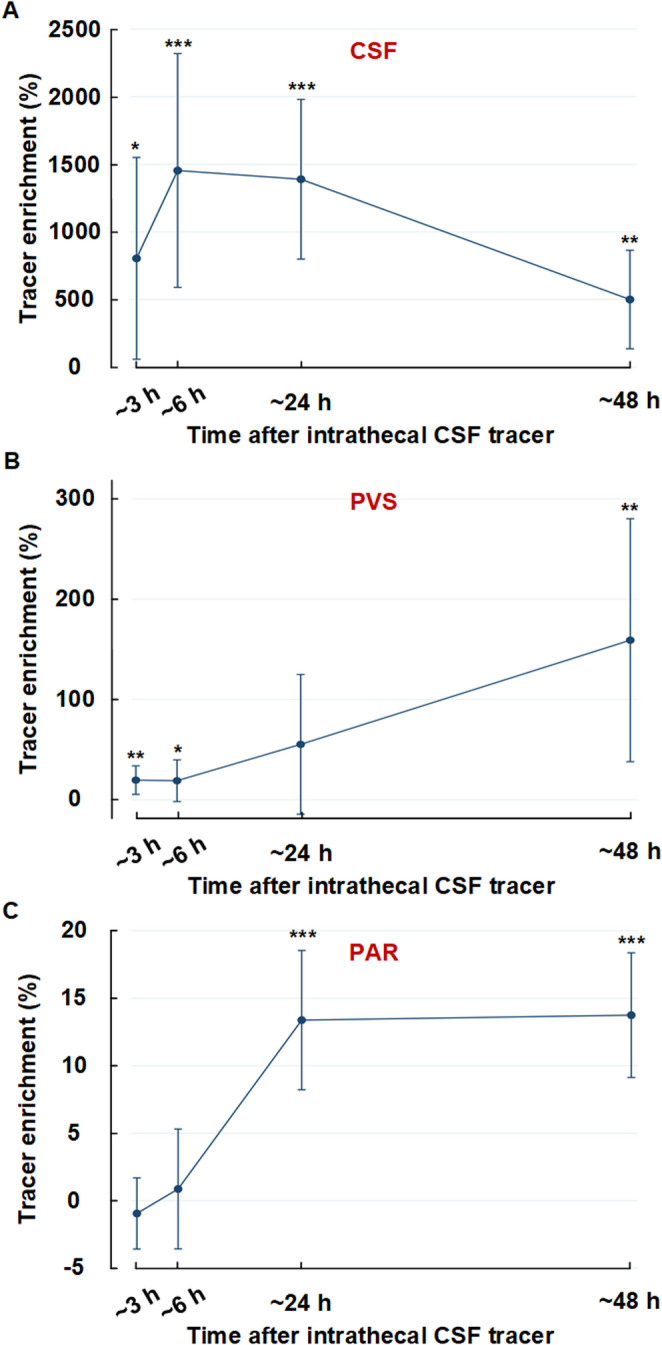
Enrichment of CSF tracer in subcortical white matter from subarachnoid CSF space. Tracer enrichment in subcortical white matter with regions of interest in (**A**) CSF nearby cortical surface, (**B**) perivascular spaces (PVS) within subcortical PVS and (**c**) brain parenchyma (PAR) nearby subcortical PVS. The significant differences in tracer enrichment as compared to pre-contrast are indicated: ^*^P<0.05, ^**^P<0.01, ^***^P<0.001 (mixed model analysis). CSF tracer enriched in subcortical PVS before in adjacent parenchyma, indicating convective transport from surface along perivascular conduits, and not diffusion via parenchyma. While tracer enhancement peaked in subarachnoid CSF after 6 hours (h), enhancement in PVS of subcortical white matter peaked at 48 hours, the latter indicating that CSF tracer accumulates in PVS of subcortical white matter, while subarachnoid CSF is far into clearance phase. Note that contrast enrichment in nearby CSF is higher in the region of basal ganglia compared to the white matter especially during the first 24 h. This can be explained by early arrival of the contrast from the spinal canal to the basal cisternal space from where it moves towards convexities. Later, this difference is less pronounced due to more proportional distribution and elimination of contrast from the CSF

**Fig. 4:**
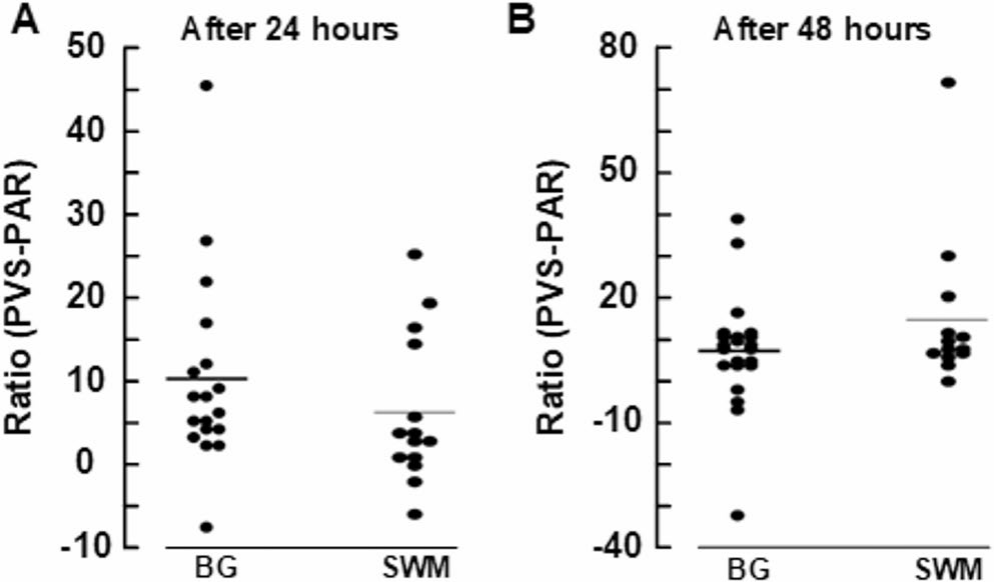
Tracer enrichment in perivascular space (PVS) versus nearby brain parenchyma (PAR). The relative tracer enrichment was expressed as the ratio of mean tracer enrichment in PVS versus nearby brain parenchyma (PAR) either (**A**) 24 hours or (**B**) 48 hours after intrathecal tracer injection. BG: Basal ganglia. SWM: Subcortical white matter. Horizontal lines refer to mean values. Given a water fraction in PVS of~100 %, and a water fraction in a brain tissue of ~20%, a ~5 times higher T1 signal in PVS implies equal concentration in the two compartments. At 24 hours, a mean ratio exceeding 5 in basal ganglia suggest they enhance more than adjacent brain tissue. In PVS of SWM, however, a ratio close to 5 suggest approximately equal tracer concentrations in PVS and SWM at 24 hours. At 48 hours, which is in late tracer clearance phase, the white matter PVS/SWM ratio has increased, indicating accumulation of tracer in PVS of SWM, and that clearance from adjacent SWM has occurred faster than from PVS. The large variability of ratios in both regions should be noted. Negative values are of technical nature and basically related to subtle effects of percentage enrichment in brain parenchyma (PAR) from already very low levels. We did not assess ratios at 3 hours and 6 hours, since enrichment of parenchyma at these time points was zero, or close to zero, where ratios would be less meaningful

**Fig. 5:**
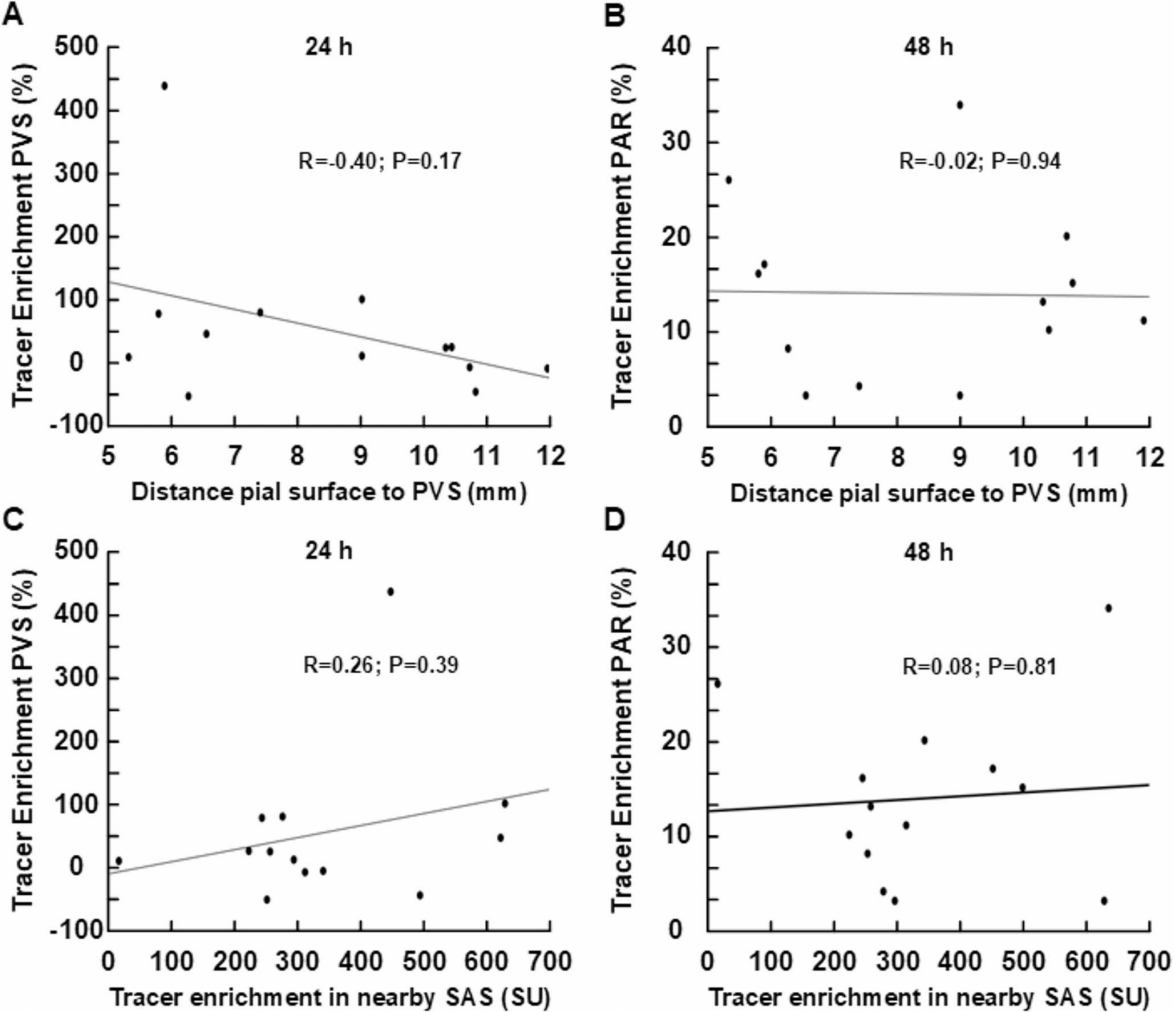
Tracer enrichment in perivascular space (PVS) and nearby parenchyma (PAR) of subcortical white matter versus depth of PVS or tracer enrichment of subarachnoid space (SAS). The correlation between the distance of PVS from pial surface and tracer enrichment in PVS at (**A**) 24 hours or (**B**) 48 hours. The correlation between tracer enrichment within SAS closest to PVS and tracer enrichment in PVS at (**C**) 24 hours or (**D**) 48 hours. Pearson correlation coefficients with significance levels are shown, including fit line. The lack of correlations, which might have been expected with tracer transport merely by diffusion in parenchyma down a concentration gradient, supports convective tracer transport in perivascular spaces

Supplementary Tables 1 and 2 present subject-specific results of CSF tracer enrichment shown as percentage T1 signal increase at 3 h, 6 h, 24 h and 48 h in CSF, perivascular space and parenchyma from both regions (basal ganglia and cerebral white matter). Table 2 provides data at group level. Figure 1 illustrated examples of tracer enrichment over time in basal ganglia and subcortical white matter. The time course of tracer enrichment is further shown graphically for basal ganglia in Fig. 2 and for subcortical white matter in Fig. 3. Contrast enrichment was detected in perivascular spaces of both the basal ganglia and subcortical white matter, demonstrating that perivascular space fluid in both regions exchange with subarachnoid CSF (Table 2; Figs. 2 and 3). There was large heterogeneity to which degree perivascular spaces enriched, particularly in cerebral white matter (see Supplementary Tables 1 to 2). Negative percentage change in tracer enrichment from baseline is per definition an artifact and can be attributed to MRI gray scale alterations that were insufficiently adjusted by normalization. Peak enhancement was reached at 6 h in perivascular space of basal ganglia with a T1 signal increase of 233 [35 to 432] % and at 48 h in cerebral white matter with a T1 signal increase of 159 [38 to 280] % (Table 2). Ratios of enhancement in perivascular spaces compared to adjacent parenchyma at each time point is shown as scatter plot for 24 h and 48 h in Fig. 4. For perivascular spaces of basal ganglia, enhancement in perivascular spaces exceeded that in adjacent parenchyma by far at both 3 h and 6 h suggesting enrichment of perivascular spaces before parenchyma (Table 2). In perivascular spaces of cerebral white matter, enrichment at 3 h and 6 h was much more subtle, but also higher than in parenchyma, which was close to zero. Here, enrichment continued to increase through the 24 hours’ time point and peaked at 48 h, where mean ratio of percentage increase in the perivascular space compared with adjacent brain parenchyma exceeded a ratio of 5, indicating tracer accumulating within the perivascular space (Fig. 4). In perivascular spaces of both the basal ganglia and cerebral white matter, a large heterogeneity of enrichment should be noted (Table 2; Figs. 2, 3 and 4). There were no correlations between CSF tracer enhancement in the subarachnoid compartment and perivascular space of subcortical cerebral white matter and basal ganglia, nor any correlations between tracer enhancement in perivascular spaces of these two regions and distance to brain surface (Fig. 5).

**Table 2:** Enrichment of a CSF tracer in perivascular spaces (PVS), nearby brain parenchyma (PAR) within basal ganglia and subcortical white matter

	Time after intrathecal tracer injection			
Basal ganglia	3 h	6 h	24 h	48 h
CSF: Percentage increase in tracer enrichment	4304 [3128 to 5481]	4737 [3624 to 5849]	1556 [933 to 2180]	337 [178 to 496]
PVS: Percentage increase in tracer enrichment	151 [−14 to 317]	233 [35 to 432]	190 [115 to 264]	131 [84 to 178]
PAR: Percentage increase in tracer enrichment	−1 [−5 to 3]	7 [2 to 12]	21 [16 to 27]	15 [9 to 21]
**Subcortical white matter**				
CSF: Percentage increase in tracer enrichment	805 [58 to 1551]	1454 [590 to 2319]	1390 [799 to 1980]	500 [136 to 865]
PVS: Percentage increase in tracer enrichment	20 [5 to 34]	19 [−2 to 40]	55 [−15 to 125]	159 [38 to 280]
PAR: Percentage increase in tracer enrichment	−1 [−4 to 2]	1 [−4 to 5]	13 [8 to 19]	14 [9 to 18]

Values presented as Mean [95% Confidence intervals in brackets]. CSF refers to tracer enrichment within subarachnoid space closest to the PVS nearby basal ganglia or subcortical white matter

## Discussion

It has been hypothesized that MRI-visible perivascular spaces in the subcortical white matter may represent an imaging biomarker of brain-wide perivascular clearance function. However, despite existing associations between MRI-visible perivascular spaces and many conditions and diseases [22], other factors than impaired clearance could cause enlargement of the perivascular spaces [23]. In this study of close to healthy human subjects, we provide evidence that MRI-visible perivascular spaces of subcortical white matter and basal ganglia enrich with a CSF tracer, preceding that of adjacent parenchyma. The higher levels of enrichment in perivascular spaces compared to in adjacent brain tissue indicate that CSF exchange with perivascular spaces in both regions occur primarily from surface along perivascular conduits, and not purely by diffusion through the parenchyma. While enrichment in perivascular spaces of basal ganglia was early, strong and highly associated with enrichment at the brain surface, enrichment of perivascular spaces in subcortical white matter was much more subtle at early time points and peaked at 48 h. A main finding of the study is the large extent of variability in perivascular space enrichment.

Subpial perivascular pathways were in animals previously shown to be a crucial constituent of CSF driven clearance of toxic metabolites from the cerebral cortex [1]. While perivascular spaces in the human brain are in general not evident at MRI, and almost never seen in the cortex, analysis of MRI-visible perivascular spaces in subcortical white matter has been proposed to possibly serve as a non-invasive imaging marker of impaired brain clearance function [10, 24, 25]. It has, however, remained unclear to what extent perivascular spaces of cerebral white matter relate to previous findings in animals where perivascular solute clearance in the cortex was imaged directly with two-photon microscopy and by that established a link between perivascular clearance dysfunction and neurodegeneration [1].

In the current study, we corroborate the recent findings by Yamamoto et al. [26], who in a study of five patients demonstrated that an intrathecal contrast agent enriched in MRI-visible perivascular spaces of subcortical white matter. The authors did not utilize images before contrast administration and assessed tracer enrichment after 24 and 48 h, respectively. From this, they concluded with proof-of-principle for a direct continuity between the subarachnoid space and PVS in subcortical white matter.

In our study, perivascular spaces of basal ganglia were used as controls. Compared to these, perivascular spaces of subcortical white matter enhanced to a much lesser degree, much later, and with larger variation between individuals. Similar to Yamamoto et al. [26], the percentage increase of normalized T1 signal units was higher in subcortical white matter perivascular spaces than in adjacent parenchyma, from which they concluded that enlarged perivascular spaces were enriched directly from surface along perivascular pathways, not via diffusion. A key to interpreting both their results and ours is that while the water fraction in an MRI-visible perivascular spaces is close to 100%, the water fraction of the extracellular space in brain tissue, which is enriched by the contrast agent (i.e. CSF tracer), is approximately 20% [27]. By assuming linearity between amount of contrast agent and T1 signal increase, equal increases of contrast agent concentrations in a perivascular space and adjacent parenchyma, respectively, would imply a T1 signal intensity increase in ratio of 5:1 (five times higher increase in the perivascular space than in the extracellular space of adjacent tissue). Yamamoto et al. [26] found higher signal in perivascular spaces than tissue at 24 h, but did not utilize scans before tracer injection, and also assessed FLAIR MRI, where the relationship between signal increase and concentration increase is less well defined. However, we believe our observations from 27 near-healthy subjects in principle add to their findings that was made in five patients.

In the current study, enrichment in perivascular spaces of subcortical white matter continued to increase at 48 h, exceeding a 5:1 ratio compared to in adjacent tissue, suggesting that tracer accumulated within the perivascular space. At the same time, enrichment in CSF at the surface had declined substantially and was thus well into clearance phase. This clearly indicates that MRI-visible perivascular spaces of subcortical white matter are dysfunctional clearance pathways. This could in theory be consistent with previous researchers` interpretation that MRI-visible perivascular spaces are dilated due to a downstream clearance obstruction in the cortex, which is without doubt the most relevant region concerning brain clearance, protein deposits and in the end neurodegenerative disease. However, the various degrees of enrichment we observed in perivascular spaces of subcortical white matter suggest MRI-visible perivascular spaces may be dilated for different reasons and may therefore not necessarily robust markers of perivascular clearance dysfunction. This is underlined by the lack of correlation of CSF tracer enhancement in CSF of the subarachnoid space and MRI-visible perivascular spaces of subcortical white matter (Fig. 5).

**Fig. 6:**
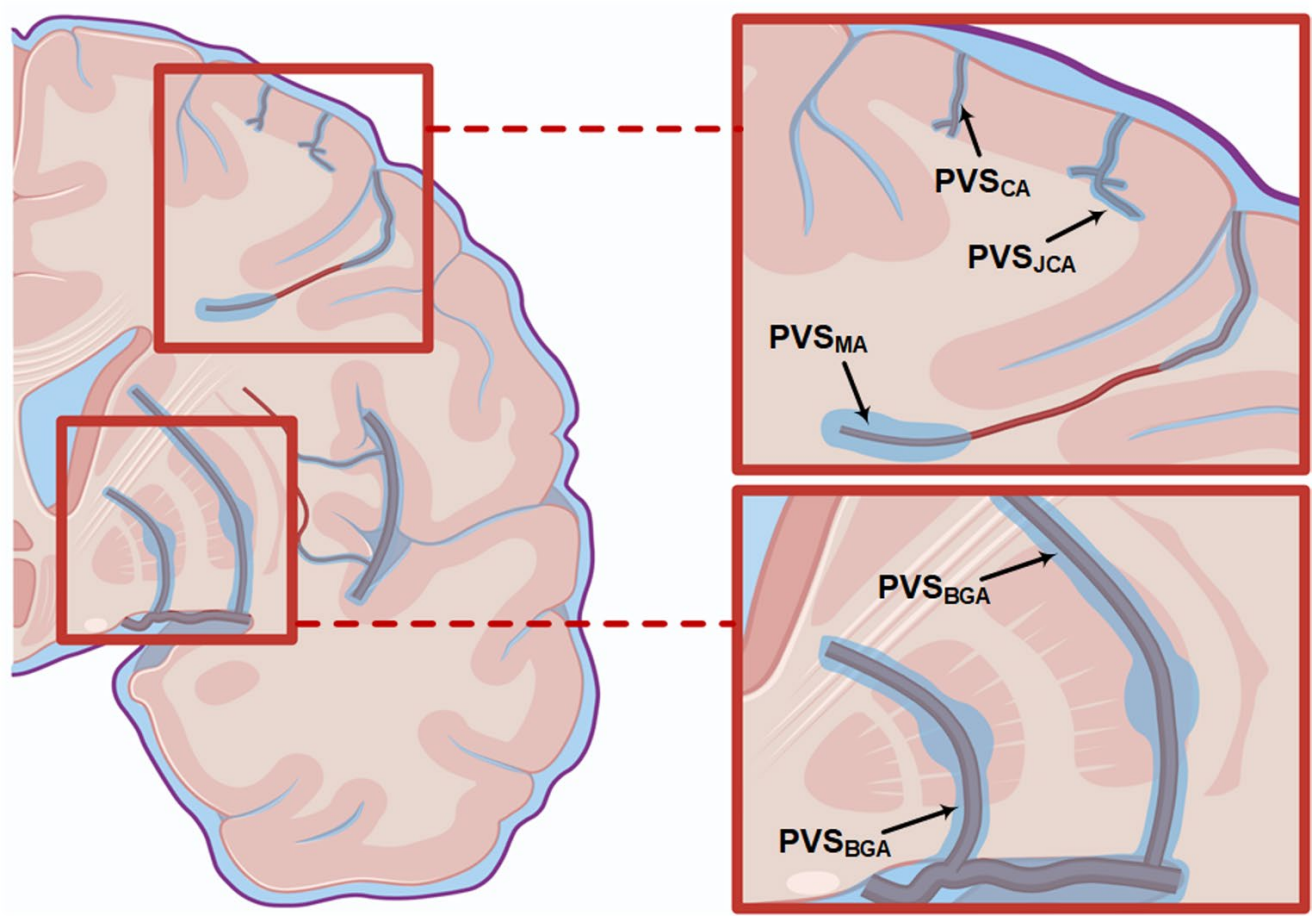
Illustration of perivascular spaces in subcortical white matter (upper right square) and basal ganglia (lower right square).For perivascular spaces along basal ganglia arteries (PVS_BGA_), anatomical studies have shown a direct connection with CSF of subarachnoid spaces [6, 31–33], which is corroborated by findings in the current study. In the cerebral cortex, anatomical studies have indicated that most penetrating arteries reside within the cortex only (i.e. cortical arteries), while some penetrate into white matter before they bend to run in parallel with the cortex in juxtacortical white matter (i.e. juxta-cortical arteries; medullary arteries are much sparser and provide blood to white matter but typically not connecting with cortical arteries [8, 34]. Perivascular spaces of subcortical cerebral white matter have previously been shown to surround (medullary) arteries [6, 7] (here denoted PVS_MA_), and are therefore not expected to connect directly to perivascular spaces of cortical arteries (PVS_CA_) or perivascular spaces of juxta-cortical arteries (PVS_JCA_). In the current study, subcortical white matter perivascular spaces (PVS_MA_) enhanced before adjacent parenchyma, supporting convective tracer transport from surface along perivascular spaces, not via diffusion in parenchyma. In these, tracer enrichment increased up to 48 hours, showing signs of accumulation when CSF and adjacent parenchyma were in clearance phase. This clearly suggests MRI-visible spaces in cerebral white matter are dysfunctional clearance pathways. However, their lack of connection with cortical perivascular spaces implicates they are dilated from other causes than downstream perivascular obstruction in the cortex, which is the main area of interest in neurodegenerative disease

Previous hypotheses about impaired clearance from MRI-visible subcortical white matter perivascular spaces are strengthened, but more likely due to impaired local clearance, and not due to downstream obstruction of perivascular clearance pathways in the cortex. In fact, the scientific basis behind the hypothesis that MRI-visible perivascular spaces are surrogate markers of brain clearance, remains unclear. Toxic protein deposits, such as amyloid-β and tau, predominantly occur in the cortex [28], which is also where a crucial role of perivascular solute clearance has been demonstrated in animal models [1, 29]. Moreover, it is well established that cortical vessels, whether arteries or veins, rarely extend into the subcortical white matter [8]. When they do, these vessels typically bend laterally to run parallel to the cortex, whereas MRI-visible perivascular spaces typically align perpendicularly to the cortex. Perivascular spaces of the white matter were previously shown to surround deep medullary arteries, which supply the outer two-thirds of the subcortical white matter and have minimal or no branching within the cortex; for a more detailed description see illustration of Fig. 6. While translational imaging research in humans plays a crucial role in bridging basic animal studies with clinical applications, it appears that the term perivascular spaces, usually abbreviated “PVS”, has been used interchangeably in referral to those of cerebral cortex, subcortical white matter and basal ganglia in parts of the imaging community, particularly perivascular spaces in the cerebral cortex are often considered as extensions of those in the subcortical white matter. Furthermore, it was also recently described in vivo a perivascular compartment surrounding arteries within the subarachnoid space, denoted the subarachnoid perivascular spaces [30]. Since perivascular spaces in various regions may have different roles, each of their location and role should be clearly separated. One way could be to differentiate between cortical perivascular spaces, generally referred to as Virchow-Robin perivascular spaces that would refer to the perivascular spaces along cortical arteries and juxta-cortical arteries, in contrast to the perivascular spaces of the subcortical white matter that refer to perivascular spaces along medullary arteries, in addition to the perivascular spaces of arteries within the basal ganglia.

As fraction of the overall brain volume, perivascular spaces, assumedly those of both grey and white matter, were estimated to constitute approximately 1%, with the vast majority being visible only under the microscope and not detectable by MRI [35]. Therefore, only a relatively minute number of perivascular spaces enlarge to the extent that they become MRI-visible, as reflected in the numbers of MRI-visible perivascular spaces that are relevant in established perivascular space-scores [18, 19]. The subjects in our study had presented with symptoms that warranted a clinical evaluation for a CSF circulation disorder. Since no CSF disturbance was found, and no treatable condition was diagnosed, we consider them therefore to be close to healthy. Their perivascular space-scores were comparable to those reported in previous studies on healthy cohorts [18, 36]. A few enlarged perivascular spaces are observed across all age groups and are typically considered a normal finding on MRI [37].

Conversely, several conditions—including advancing age, vascular dementia, arterial hypertension, and traumatic brain injury—are associated with an increased number and size of perivascular spaces [23, 24]. While these conditions are often linked to impaired brain function, our findings suggest that other confounding factors should be investigated as contributors to perivascular space enlargement in these groups. These factors may include arterial spiral elongation, local atrophy, loss of myelin, or immune cell deposition within the perivascular space [23]. Our findings that CSF tracer enrichment in MRI-visible perivascular space varies much between subjects also indicate there may be various causes behind their enlargement, making MRI based scores where all perivascular spaces are lumped together into one score, less meaningful.

### Limitations

Off label use of intrathecal MRI contrast agents at gMRI has limited the methodology from widespread application in healthy subjects and clinical cohorts, even though prospective safety studies have shown a good safety profile [13–15, 38]. On label use of intravenous contrast agent could be considered a less invasive alternative. However, since contrast agents leak from blood into the CSF as well as across the blood-brain barrier, interpretation of findings may be challenging [39]. Completely non-invasive methods, such as diffusion tensor imaging along perivascular spaces (DTI-ALPS), also strive with major validity issues [40].

Another limitation to this study is that we measured enhancement within MRI-visible perivascular spaces by a manually placed ROI. To assure robust measurements, only perivascular spaces with a diameter of ≥ 2 mm was selected. Smaller perivascular spaces, that still may be visible at MRI, are therefore not part of the analysis. Since our study subjects were close to healthy, our results may not be valid for perivascular spaces in patients with neurological disorders, which should be done in future studies. Moreover, in subjects with more than one perivascular space per region with size ≥ 2 mm, the analyzed perivascular space in each region was randomly selected. An automated segmentation algorithm may have rendered for the inclusion of more PVS from each region. A higher temporal resolution of MRI scans would have allowed for a more precise determination of CSF tracer dynamics in the various compartments.

Finally, MRI grey scale alterations during repeated MRI acquisitions represents a technical limitation; to overcome this we normalized the images against a reference region and assessed percentage change in tracer enrichment, as compared to pre-contrast. Our findings of negative percentage tracer enrichment at early time points in some subjects, indicates this normalization is not perfect. In future studies, quantitative T1 with a similar image resolution to the one in our current study, would be preferrable.

## Conclusion

This study provides evidence that CSF tracer enters MRI-visible perivascular spaces from the subarachnoid space via perivascular conduits of medullary arteries, with subsequent accumulation in these spaces. Both basal ganglia and subcortical white matter perivascular spaces demonstrate tracer enrichment, but with considerable heterogeneity. In white matter, PVS enhance more slowly and less intensely than surrounding parenchyma, with peak enhancement occurring as late as 48 h, suggesting accumulation rather than effective clearance.

The marked variability in tracer enrichment across perivascular spaces complicates the interpretation of their enlargement as a consistent marker of impaired waste clearance. Moreover, anatomical studies indicate that white matter perivascular spaces, which ensheath medullary arteries, are not directly connected to the perivascular clearance pathways responsible for waste removal from the cortex. This anatomical distinction casts further doubt on the assumption that MRI-visible PVS in cerebral white matter reflect global brain clearance function.

Taken together, our findings question the validity of using MRI-visible white matter perivascular spaces as surrogate biomarkers for brain-wide clearance dysfunction. Their slow and heterogeneous tracer dynamics suggest local accumulation, but likely not as a direct consequence of upstream failure in cortical perivascular clearance pathways.

## Supplementary information

Below is the link to the electronic supplementary material.

Supplementary Material 1

## Data Availability

The data presented in this work is available on reasonable request.
