# Supplementary material for: Do enlarged white matter perivascular spaces reflect brain clearance dysfunction? Insights from intrathecal contrast-enhanced MRI
Source: Neuroradiology. 2025 Dec 26;68(1):241–51. doi: 10.1007/s00234-025-03877-7 (PMC12906596; doi:10.1007/s00234-025-03877-7)
Supplement: Supplementary file 1 — Supplementary Material 1 [file 234_2025_3877_MOESM1_ESM.pdf]

Supplementary material

Do Enlarged White Matter Perivascular Spaces Reflect Brain Clearance Dysfunction? Insights from Intrathecal Contrast-Enhanced MRI

Supplementary Figure 1

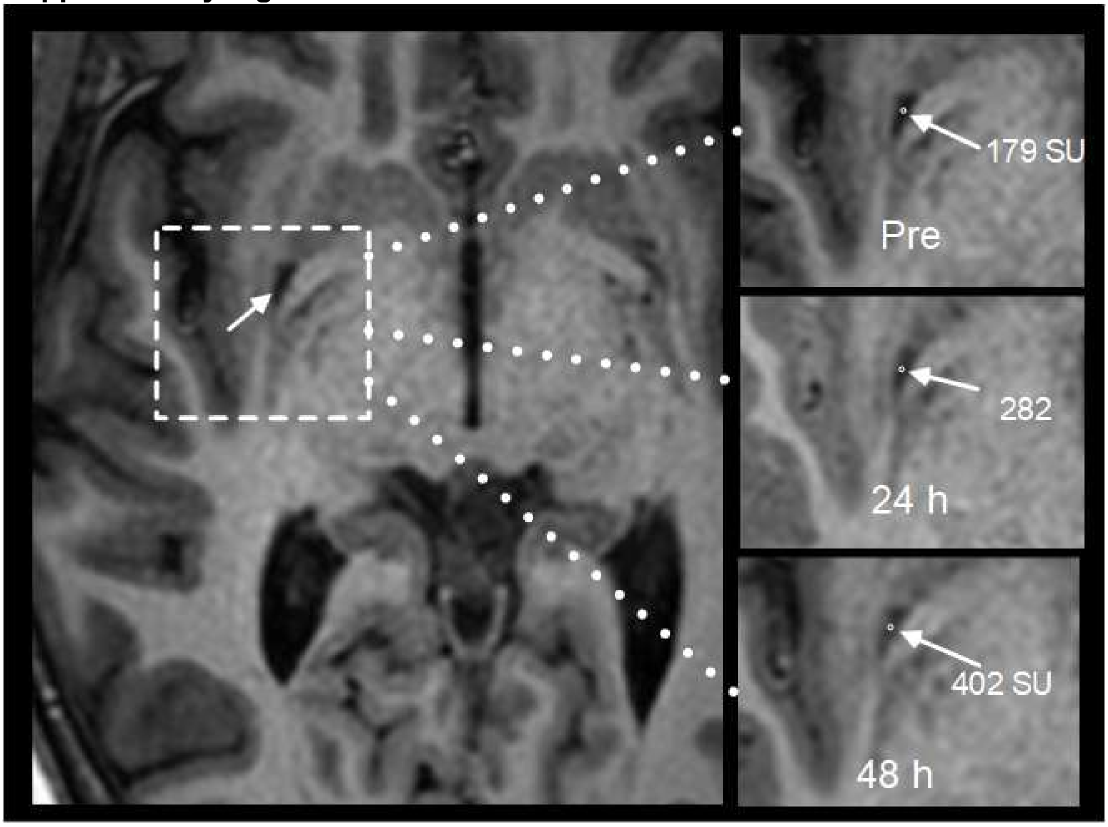

T1-weighted image of basal ganglia. MRI-visible PVS is pointed with arrow. To the right: zoomed T1 images of MRI-visible PVS with and without assessment of the signal intensity change. Images are obtained before contrast application(left), 24h(middle) and 48h(right) after contrast application.

Supplementary Figure 2

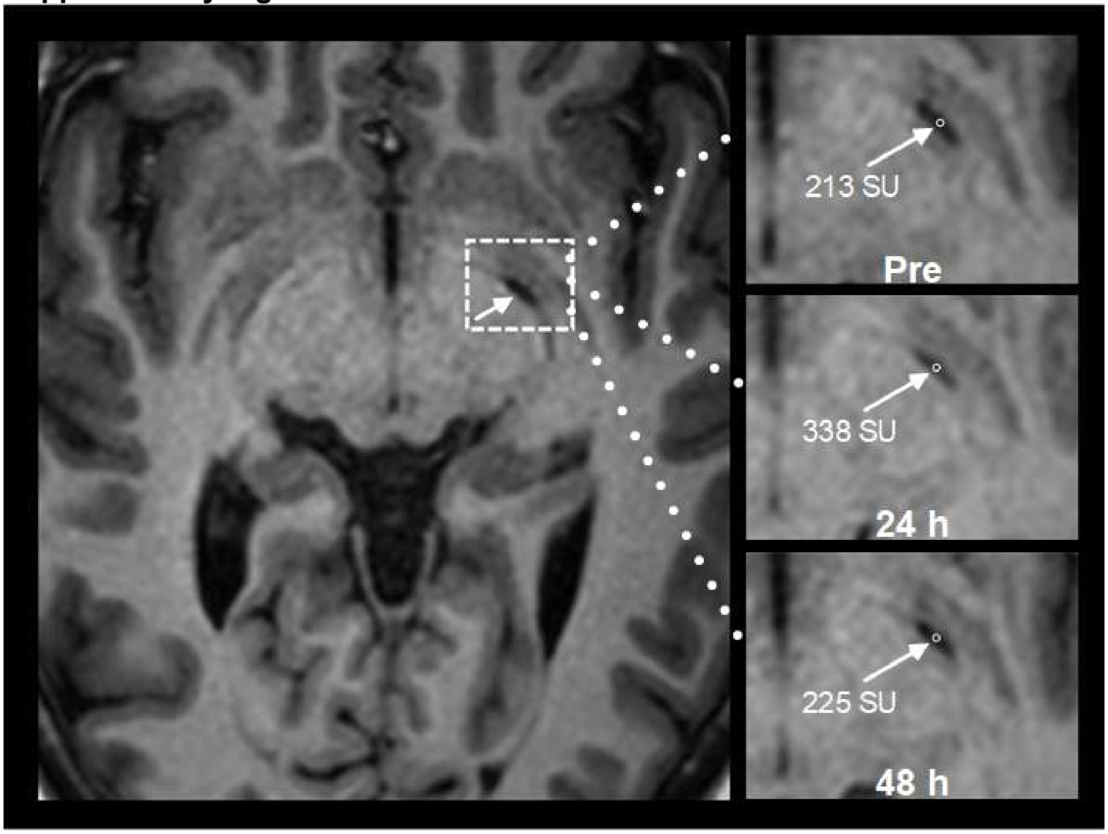

T1-weighted image of basal ganglia. MRI-visible PVS is marked with stippled square. To the right zoomed T1 images of MRI-visible PVS with assessment of the signal intensity change: before contrast application, at 24 and 48h after contrast application.

Supplementary Figure 3

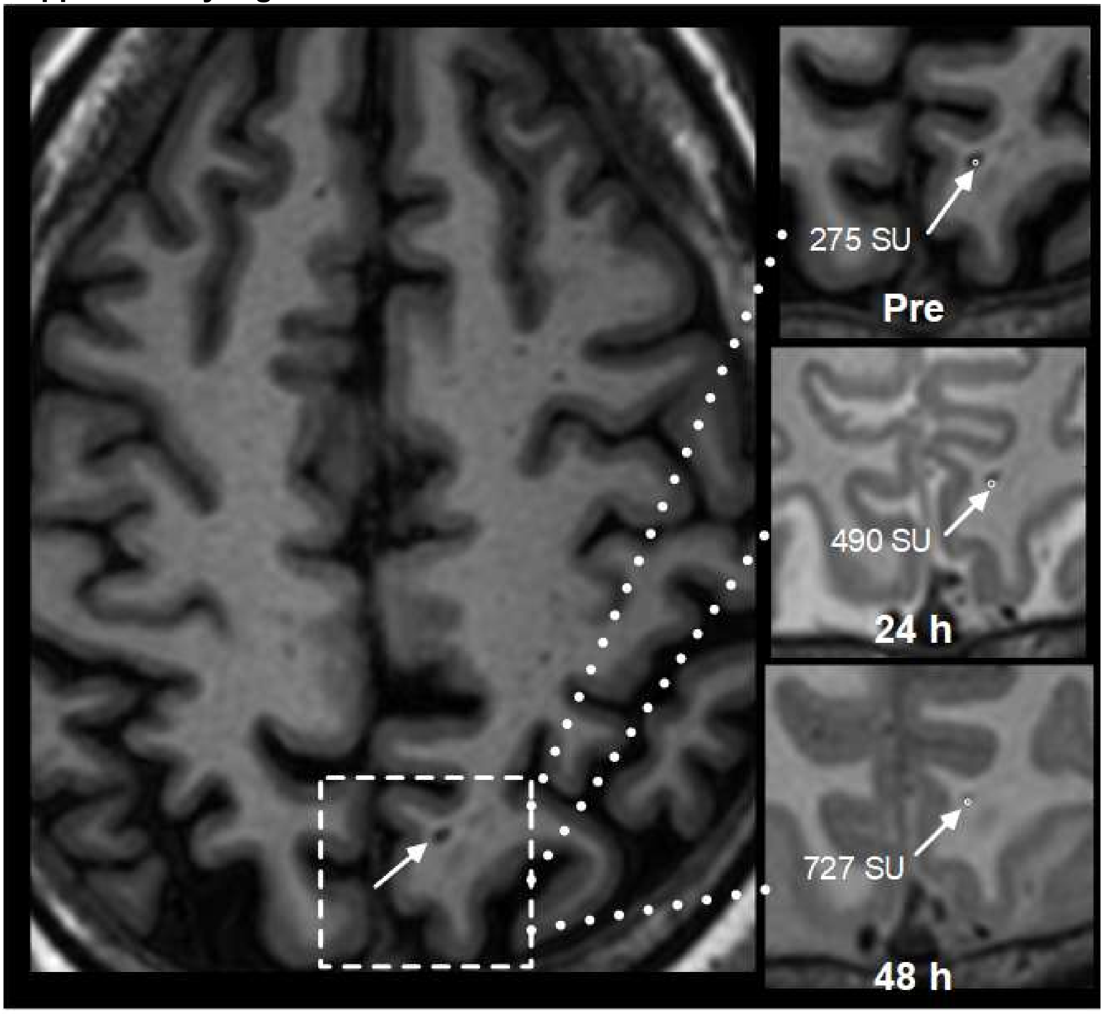

T1- weighted image of subcortical white matter. MRI -visible PVS is pointed with arrow. To the right: zoomed T1 images of MRI – visible PVS with and without assessment of the signal intensity change. Images are obtained before contrast application (left), 24h(middle) and 48h(right) after contrast application.

Supplementary Figure 4

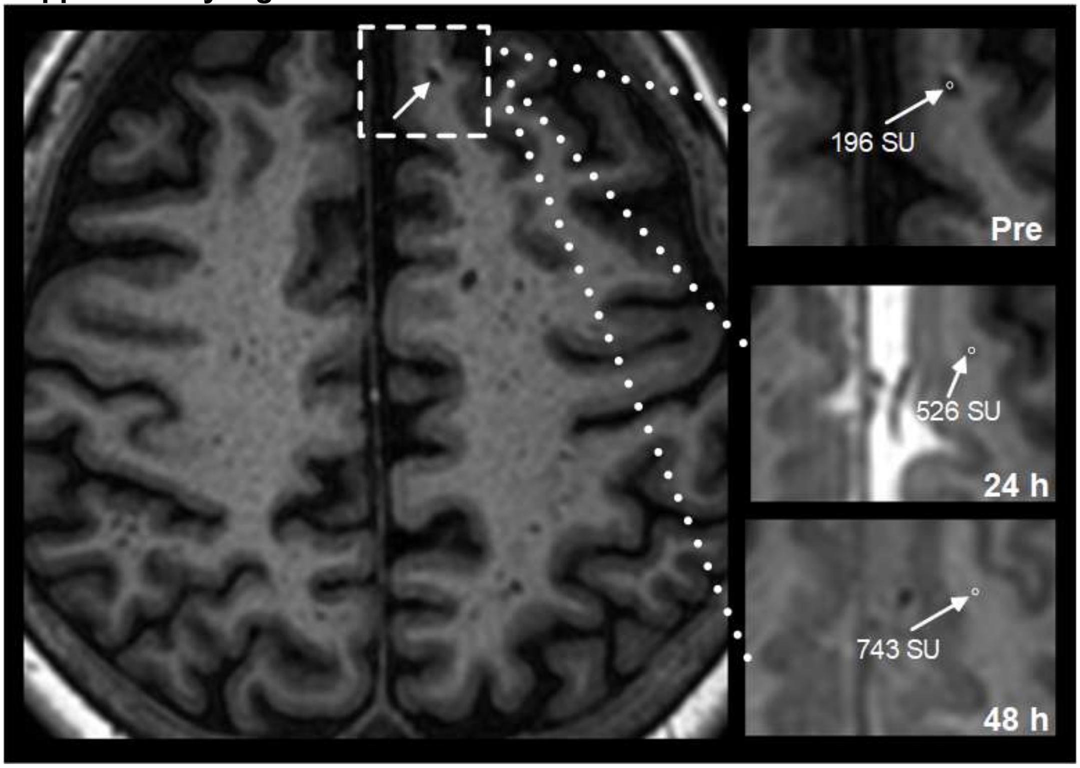

T1- weighted image of subcortical white matter. MRI -visible PVS is marked with stippled square. To the right zoomed T1 images of MRI – visible PVS with assessment of the signal intensity change: before contrast application, at 24 and 48h after contrast application.

Supplementary Table 1. Percentage increase in tracer enrichment of basal ganglia perivascular spaces and nearby parenchyma and cerebrospinal in nearby subarachnoid space

	Basal ganglia											
	Perivascular spaces				Brain parenchyma				CSF in nearby subarachnoid space			
**Patient**	**3 h**	**6 h**	**24 h**	**48 h**	**3 h**	**6 h**	**24 h**	**48 h**	**3 h**	**6 h**	24 h	48 h
**1**		-30	55	46		-1	12	-6		7775	1583	229
**2**												
**3**												
**4**	37	6	96	81	-7	-7	10	23	719	2830	2273	793
**5**	1706	2092	759	315	-24	24	34	34	6712	6670	2068	170
**6**	26	182	300	42	2	27	17	-1	4475	4777	955	226
**7**	-11	4	146	238	-2	3	32	24	12660	13404	6689	1359
**8**	407	388	111	118	0	-3	4	4	1774	1807	618	109
**9**	8	71	137	204	-1	-7	32	20	7841	7867	4185	1206
**10**	48	247	528	445	4	0	12	11	3308	3993	1570	508
**11**	19	38	83	82	-8	11	13	25	788	2592	589	238
**12**	-5	-41	57	31	10	11	30	11	2380	2717	40	-22
**13**												
**14**	23	79	160	43	-3	-5	21	6	1724	2593	877	87
**15**	119	664	193	80	0	16	23	11	7133	7332	1450	644
**16**	41	27	29	73	-3	6	6	-1	4942	4705	629	108
**17**	-7	203	197	74	-14	29	16	9	4423	3384	236	205
**18**	-24	-2	58	-16	21	20	25	6	2529	2848	701	48
**19**	-11	-31	121	133	-1	-4	45	44	3093	3426	785	35
**20**	111	69	35	8	-6	-12	-5	-1	3697	2551	333	105
**21**	125	308	379	277	7	3	34	26	4551	4304	1901	236
**22**												
**23**	532	773	265	112	-1	5	23	11	4321	4608	1456	156
**24**	78	211	177	143	-3	0	13	13	4605	4871	1396	54
**25**	21	125	60	135	13	9	27	37	4321	4411	1114	181
**26**	52	-4	201	194	7	17	27	27	3087	3599	1803	477
**27**	34	-15	213	154	-5	10	36	10	5609	5875	2547	600
**Average**	**151**	**233**	**190**	**131**	**-1**	**7**	**21**	**15**	**4304**	**4736**	**1556**	**337**

Supplementary Table 2. Percentage increase in tracer enrichment of subcortical white matter perivascular spaces and nearby parenchyma and cerebrospinal in nearby subarachnoid space

	Subcortical white matter											
	Perivascular spaces				Brain parenchyma				CSF in nearby subarachnoid space			
**Patient**	**3 h**	**6 h**	**24 h**	**48 h**	**3 h**	**6 h**	**24 h**	**48 h**	**3 h**	**6 h**	**24 h**	**48 h**
**1**												
**2**												
**3**												
**4**	5	-5	22	47	5	-3	13	13	-16	632	1020	465
**5**	40	17	76	80	-1	1	16	16	25	504	592	169
**6**	47	92	101	61	-2	18	7	-1	413	785	366	63
**7**	40	37	99	211	0	13	34	26	-83	1288	1458	210
**8**												
**9**	-16	5	44	182	-3	-5	3	10	19	-2	3177	2283
**10**												
**11**												
**12**	34	-17	7	55	-7	6	26	12	35	63	-26	0
**13**												
**14**	1	-6	-55	-21	6	2	8	10	11	2589	1318	275
**15**												
**16**												
**17**	12	18	9	37	0	-3	3	6	1246	747	324	187
**18**												
**19**	-26	70	23	37	-12	-6	10	20	853	1531	409	63
**20**												
**21**	-1	4	-9	166	1	3	20	18	1619	2213	1743	272
**22**												
**23**												
**24**	30	13	438	819	-2	-9	17	28	-34	86	1587	377
**25**	60	61	78	329	-1	4	4	5	3203	4413	1861	456
**26**	24	-43	-48	114	3	-6	15	18	45	931	2709	1447
**27**	23	22	-11	111	1	0	11	11	3929	4580	2919	738
**Average**	**20**	**19**	**55**	**159**	**-1**	**1**	**13**	**14**	**805**	**1454**	**1390**	**500**

CSF: Cerebrospinal fluid
